# Mini review on collagens in normal skin and pathological scars: current understanding and future perspective

**DOI:** 10.3389/fmed.2024.1449597

**Published:** 2024-07-18

**Authors:** Claire Jing Zhou, Yuan Guo

**Affiliations:** ^1^The Grammar School at Leeds, Leeds, United Kingdom; ^2^School of Food Science and Nutrition, University of Leeds, Leeds, United Kingdom

**Keywords:** collagen, structure and assembly, cytokine, inflammation, scar

## Abstract

Pathological scar tissues are characterized by the presence of overabundant collagens whose structure and organization are also different from those in unwounded skin. This causes scar tissues to lose some functions performed by normal skin, and currently, there are no effective measures to prevent scar formation. Inflammation has been shown to modulate fibroblast proliferation, differentiation, and function, hence collagen production and organization. In this minireview, we provide an overview of the current understanding of collagen, specifically collagen type I and III which are main collagens in skin, structure and fibre formation and highlight their differences between normal skin and pathological scars. We discuss the role that cytokines play in modulating fibroblast function. We also identify some potential research directions which could help to further our understanding of the complex and dynamic wound healing and scar formation process.

## 1 Introduction

Skin is the largest organ in the human body and acts as a physical barrier to prevent harmful substances from entering the body, protecting the body against trauma and sensing stimuli. It consists of three layers: the innermost layer, the hypodermis, comprises loose areolar and adipose tissue which connect the skin to other structures to provide insulation and cushioning through fat storage. Above that lies the dermis which accounts for the majority of skin thickness at 3–5 mm and contains extracellular matrix (ECM) proteins, mainly collagens, and fibroblasts which produce ECMs. Collagens form well-organized networks to provide overall strength and integrity to the skin (1). The outermost layer, the epidermis, is the thinnest layer of skin but contains layers of different cells and proteins. Dead keratinocytes, hardened protein (keratins), and lipids form a protective barrier on the surface to seal the skin off from the outside environment.

Due to its exposure to external threats such as physical trauma, including wounds and burns, skin tissue can often get injured. Following injury, it undergoes a wound-healing process (2, 3) which starts with rapid clot formation which covers the injured site, preventing pathogen invasion. Immune cells are recruited to clear dead cells and fight against microbes, which is crucial to prevent the chronicity of inflammation. Many signaling proteins, mainly pro-inflammatory cytokines, are also produced which further recruit fibroblasts to the site and instruct them to proliferate and differentiate to produce collagen and other ECM components. In the late stages of wound healing, anti-inflammatory cytokines become predominant and excessive immune cells and fibroblasts undergo apoptosis. Deposited collagens undergo procession and reorganization, aiming to resolve to their unwounded state, however, in adult skin, this does not lead to 100% restoration and scar tissues form. Scars are characterized by excessive collagen deposition and altered collagen fibril structure and network arrangement (4, 5). In a hypertrophic scar, dense and disorganized collagens fill the wound site, but they expand beyond this in a keloid scar. This not only causes cosmetic deformities but also reduces skin flexibility and strength resulting in discomfort, pain, and even long term disability, which can dramatically affect a patient’s quality of life (6–8). It is estimated that clinical management of skin scarring costs the National Health Service (NHS) £8.3 billion annually in the UK alone (9).

Pathological scars have been shown to be caused by prolonged inflammation which leads to inappropriate fibroblast function during wound healing. Here, we review the current understanding of skin collagens, specifically type I and type III collagen structure and assembly, and highlight their differences in normal skin and pathological scars. We discuss the contribution of cytokines in scar formation through their modulation of fibroblast functions. We identify some research gaps to further our understanding of scar formation, and which can potentially be targeted for novel treatment development.

## 2 Collagen in normal skin and scars

Collagen comprises about three quarters of the dry weight of human skin, and is the most prevalent component of the ECM (10, 11). The collagen family consists of 28 different members, (10, 12) however, collagen type I and type III are the main ones found in skin, constituting roughly 80–85% and 8–11% of the dermal ECM, respectively (13).

Each collagen molecule is a trimeric protein that consists of 3 parallel polypeptide chains intertwined together to form an elongated structure (10, 12). The collagen helical region contains a repetitive Gly-X-Y sequence, where X and Y can be any amino acid residue, but are frequently proline and hydroxyproline (10–13). This specific sequence allows three polypeptide chains to pack tightly together (10). Even though both collagen I and III share a high amount of amino acid sequence similarity, collagen III actually contains 5 more Gly-X-Y units in its helical region than collagen I (14).

**Figure 1:**
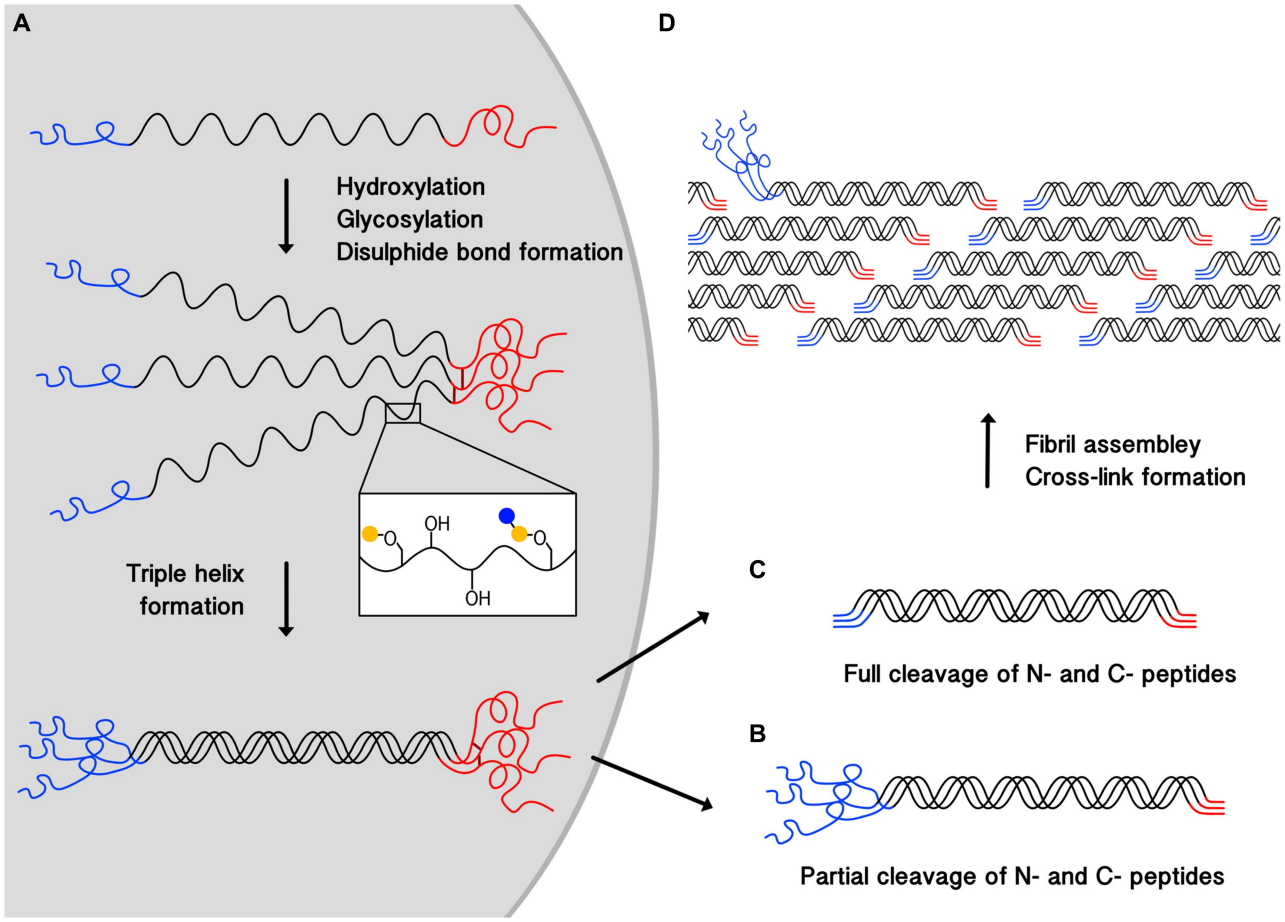
Schematic illustrating the process of collagen triple-helix and fibril formation. **(A)** Shows the synthesis of procollagen, post-translational modification, where blue and yellow circles represent monosaccharides, disulphide bond (dark red lines) formation between the C-termini (red), and the helix propagation towards the N-termini (blue). Cleavage of N and C-terminal propeptides occurs outside the cell. **(B)** Shows a partially processed pN-collagen where only C-terminal propeptide is cleaved. **(C)** Shows a fully processed collagen where both termini are cleaved. **(D)** Demonstrates collagen fibril assembly and cross-linking formation, partially cleaved pN-collagens are found on the fibril surfaces. pN-collagens contain unstructured extra propeptides which prevent them from being fully incorporated into the center of the structurally well-organized collagen fibril.

All types of collagen are initially synthesized as a procollagen chain with N- and C-terminal propeptides flanking the collagen helical region (12) (Figure 1). The procollagen is then modified through the hydroxylation of proline and lysine residues, and glycosylation of selected hydroxylysine residues through enzyme-catalyzed reactions. Hydroxylation increases the thermal stability of collagen, and some hydroxylated residues are later further oxidized outside the cell, leading to collagen cross-linking which stabilizes assembled structures. The role of glycosylation in collagen stability is not fully clear.

The triple helix formation starts with C-terminal propeptide interactions, leading to the alignment of the three chains, and the inter-chain disulphide bond formation there further ensures they are in register (10). The triple helix propagates toward the N-termini (10, 11).

After trimeric structure formation, procollagens will be secreted outside the cell where C- and N- terminal propeptides will be cleaved. Resulting collagen type I and III molecules are both around 300 nm long (12) with a diameter of 1–2 nm (11, 12). But in the adult dermis, the N-termini propeptides (pN) in type III collagen, which contain 129 amino acids, are often only partially processed, leaving the pN-collagen type III to be deposited at detectable levels (15, 16).

In skin, collagens further assemble into collagen fibrils, and collagen type I and III can also co-assemble to form hybrid fibrils with diameters above ~100 nm (11, 12, 16, 17). Fibrils are formed by collagen molecules arranging into a linear staggered array (11–13, 18) (Figure 1). Lysine and hydroxylysine are also oxidized by lysyl oxidases (LOXs) (10, 11, 18–20) to form covalent bonds both between subunits within a collagen molecule (intra-molecular cross-linking), and between different collagen molecules (inter-molecular cross-linking) (10, 18, 21). Cross-linking stabilizes fibril structures, contributing to their stiffness and mechanical resilience, (11, 22) which provides the skin with mechanical strength and stability by resisting deformation under external stress (10, 21).

It has been suggested that in hybrid fibrils, collagen type I forms the bulk of the fibrils while collagen type III only forms a coat on the surface (23). However, a recent X-ray diffraction study on the colonic submucosa of rats showed that the positions of type I and III collagens in the fibril could be random (17). The discrepancy here can be attributed to the processing of procollagen type III, which takes longer than the N-terminal cleavage of procollagen type I (24). The presence of the N-termini non-collagen region prevents pN-collagen type III from being incorporated into the centre of the fibril effectively (24) (Figure 1). This could also explain the role collagen type III plays in modulating collagen I fibrogenesis and controlling fibril diameter (16, 17, 25), as the attachment of pN-collagen type III will cause steric hindrance, thereby preventing the fibril from growing further laterally.

In skin, collagen fibrils can further assemble into fibres and bundles whose diameters lie in the micrometre range (11), they then further pack closely to form ordered networks. It has been proposed that these are lattice-like plane structures, with collagen fibres lying parallel to the epidermis, with no out-of-plane components. Whilst a histological study and a recent study using combined multiphoton imaging showed that the majority of collagen fibres in the dermis run parallel, some fibres were found to orient out of the plane (26–28).

Collagens in pathological scars show a different structure and assembly. A recent Transmission Electron Microscopy (TEM) imaging on collagen fibril ultrastructure showed that the average collagen fibril diameter was significantly reduced in keloids (~76 nm) compared with that in normal skin (~124 nm). The average collagen fibril diameter was also found to be smaller in hypertrophic scars compared to normal skin (29–31). In mice, it was found that collagen fibrils after 14 days of healing had around half the diameter of those found in unwounded tissue (29).

SEM images of hypertrophic scars showed that the majority of fibre bundles are loosely arrayed in a wavy pattern that runs parallel to the epithelium, whereas in keloids, bundles are packed loosely with a random orientation (18, 32). Moreover, keloid collagen fibres are thicker than those of normal skin and hypertrophic scars (32, 33).

The relative ratio of type III to type I collagen was found to be reduced in pathological scars compared to the unscarred adult dermis (34, 35). However, it is not clear why the diameter of scar fibrils was found to be thinner, not thicker as expected.

Hydroxylation of type I collagen was also found to be significantly higher in keloids, and LOX activity is elevated in pathological scars compared to normal skin (18, 36). These lead to excessive collagen cross-linking which is consistent with the thicker fibres observed. Table 1 summarizes the differences of collagen in normal skin and pathological scars.

**Table 1:** Comparison of the collagen composition, assembly, and arrangements in normal skin and pathological scars.

	Normal skin	Hypertrophic scar	Keloid
Average collagen fibril diameter (nm)	~110–130 in dermis (29–31)	~60 (30)	~60–70 (30, 31)
Collagen fibre orientation	Mainly parallel to the dermis surface with minor being oriented out-of the plane (27, 28)	Mainly parallel to epithelia surface (18, 32)	Oriented randomly to the epithelial surface (18). Irregular shape and unevenly spaced as compared to normal skin nearby (37)
Thickness of collagen fibre compared to normal skin	/	Finer fibers (33) Thinner than normal skin (32)	Thicker bundles than normal skin and hypertrophic scar (32, 33)
Packing of collagen fibers	Majority of fibres lie in a parallel array and closely packed (18, 28)	Loosely arrayed in a wavy pattern, more fragmented and shorter than normal (18)	Packed loosely (18)
Lysyl oxidase (LOX) activity compared to normal skin	/	Comparable activity to normal skin (18)	Higher LOX activity (~3 times) than normal skin
mRNA ratio of procollagen type I/III in fibroblasts	5.2 (normal fibroblast) (35)	/	22.1 (pathological fibroblasts) (35)
Ratio of collagen type I/III expression in fibroblasts	6.3	7.7	17.3 (34)

## 3 Cytokine modulation of fibroblast collagen production and assembly

Fibroblasts and myofibroblasts (collectively called (myo)fibroblasts) are responsible for ECM, and therefore collagen type I and III, production and deposition during wound healing (38). Fibroblasts migration and differentiation into myofibroblasts, which are also described as activated fibroblasts, is modulated by many factors including mechanical tension and cytokine signaling. Myofibroblasts are characterized by the expression of α-smooth muscle actin (α-SMA) stress fibres, which generate high contractile forces for wound contraction (39–41). Apart from collagen production, (myo)fibroblasts also modulate collagen properties through synthesizing enzymes such as LOXs, which catalyze collagen cross-linking in fibril formation. During normal scar formation, myofibroblasts will undergo apoptosis at the later stages of wound healing to prevent excessive collagen synthesis, however, they are found to be resistant to apoptosis in pathological fibrotic tissues which can promote fibrosis and scar formation (42). Some cell based studies do not differentiate between fibroblasts and myofibroblasts, and refer to both as fibroblasts.

It has been shown that pro-inflammatory cytokines stimulate fibroblast proliferation, differentiation and function, hence collagen production and assembly, however, the research results obtained can be controversial. Interleukin-1 (IL-1) is often categorized as pro-inflammatory cytokine, and its concentration is found to increase when the skin is injured (6, 43). It has two forms: IL-1α and IL-1β, which both can bind to the same IL-1 receptor type (IL-1R1) expressed on the surface of fibroblasts. Successful IL-1 signaling requires its binding to the third extra-cellular immunoglobulin domain of IL-1R1 and also a second receptor chain, termed either IL-1R accessory protein (IL-1RAcP) or IL-1R3, forming a trimeric signaling complex (44, 45). IL-1β can also bind to IL-1R2, a decoy receptor belonging to the IL-1R family (44, 45) which shares a very similar architecture with IL-R1, but which possesses only a very short intracellular segment (46), hence its binding inhibits the pro-inflammatory activity of IL-1β (45). Therefore, the function of IL-1β and IL-1α in stimulating fibroblast differentiation and collagen deposition can be different.

Early studies using cultured dermal fibroblasts showed that recombinant IL-1α or IL-1β stimulation resulted in cell proliferation and enhanced collagen production (47). The purified human IL-1 stimulation of cultured normal human dermal fibroblasts resulted in a nearly doubled production of collagen type I and type III compared to those without stimulation. Interestingly, a similar test using IL-1 on scleroderma fibroblasts from both affected and unaffected skin areas did not produce any obvious responses (48). However, in a separate study, recombinant human IL-1α or IL-1β showed decreased collagen type I and III accumulation by fibroblasts, although the mRNA levels of both collagens were found to have increased (49). These contradictory results make it difficult to determine the functions of IL-1α and IL-1β. The source of fibroblasts and different experimental conditions, such as the different bovine serums used to grow fibroblasts, could have affected fibroblast responses.

Despite these contradicting results, animal studies targeting IL-1 levels have been shown to inhibit pathological scar formation. The occlusion with silicone gel of a rabbit ear wound was found to reduce the expression of IL-1, and collagen deposition, inhibiting fibrosis and hypertrophic scarring (50). Curcumin treatment has been shown to significantly reduce hypertrophic scarring in New Zealand White rabbits, which was associated with a significant decrease in the production of pro-inflammatory cytokines including IL-1 and IL-6 (51).

IL-6 is another major regulator of the acute inflammatory response (52, 53). It signals by binding to an IL-6 receptor (IL-6R), and the IL-6/IL-6R complex is then associated with gp130, a protein expressed on all cells (54), to initiate intracellular signaling via the JAK/STAT pathway (54). IL-6, IL-6R, and gp130, are greatly elevated in keloid fibroblasts compared to those in normal skin (53). The effects of IL-6 include directly stimulating the migration of fibroblasts to sites of injury, increasing type I collagen expression and synthesis, and/or the production of transforming growth factor β (TGF-β) (52) which plays a very important role in the proliferation of fibroblasts, and their further differentiation into myofibroblasts (43). The inhibition of STAT3 expression to block IL-6’s signaling pathway or inhibit IL-6 in keloid fibroblast cultures has resulted in keloid tissue fibroblasts losing collagen production, impairing their proliferation (40, 55). IL-6 may also influence myofibroblast persistence, due to its ability to induce Bcl2 expression in fibroblasts, which has an anti-apoptotic signaling function (53). Myofibroblasts present in hypertrophic scars also were found to be resistant to apoptosis (56).

However, IL-6 can also exert anti-inflammatory activities (57). Later in the wound healing process, the expression of IL-6 is significantly decreased (53) and IL-6 and IL-10 (an anti-inflammatory cytokine) promote macrophage polarisation from the pro-inflammatory M1 phenotype to the anti-inflammatory M2 phenotype (53, 58). This switches off inflammation by enhancing secretion of anti-inflammatory cytokines such as IL-10 and growth factors such as TGF-β (53, 59). It seems concentrations of cytokines, and their environment modulate fibroblast responses but the underlying mechanism is unclear.

Despite its potential anti-inflammatory effects, treatments for pathological scarring mostly involve inhibiting IL-6’s pro-inflammatory functions (52). The intralesional injection of corticosteroids causes scar regression by reducing IL-6 expression (50). Corticosteroids have been shown to supress the inflammatory process in the wound (60), inhibit fibroblast growth (6, 61) and increase the activity of collagenase (62), resulting in the degradation of collagen in scars (6, 61, 62). Pirfenidone, an FDA-approved drug used to treat fibrotic disorders, has been shown to reduce IL-6 and other pro-inflammatory cytokines in the wounds of treated mice during the inflammatory phase of deep burn wound healing (63).

The transforming growth factor β (TGF-β) family contains 33 known proteins in humans (64) and are bifunctional regulators that can either inhibit or stimulate cell proliferation (43) and is involved in all stages of wound healing (65). Among them, three isoforms, TGF-β1, −β2, and -β3, are the best-studied ones (64). The canonical pathway for TGF-β involves the Smad family of transcriptional activators, which function to transmit TGF-β stimulations to the nucleus. Signaling through the canonical ALK5/Smad3 pathway is crucial for the pathogenesis of fibrosis in many tissues.

TGF-β1 directly promotes myofibroblast development by inducing the expression of α-SMA and ECM protein production (59, 66–68). Its overexpression induces and sustains the activation of keloid fibroblasts (43). The administration of TGF-β1 to normal and keloid fibroblasts was found to dramatically increase the levels of intracellular collagen type I and III in both cells (69). Moreover, the addition of TGF-β1 to foetal wounds (which are typically scar-free) has been shown to induce scar formation (68).

TGF-β2 also recruits fibroblasts to the wound site, resulting in increased collagen deposition (particularly type I and III) during matrix formation, which can lead to scar formation (43). When TGF-β1 and TGF-β2 were blocked with neutralising antibodies, the severity of scarring was found to be significantly decreased, further implicating their roles in scar formation (70).

Unlike TGF-β1 and TGF-β2, TGF-β3 inhibits scarring by limiting the inflammatory reaction and has been shown to reduce scar formation and promote better collagen organization when injected into adult wounds during clinical trials, and reduce scar formation in animal models (60, 65, 71–73). It is highly expressed in foetal wounds, which may act as a key contributor to the scarless healing phenotype. The ratio of TGF-β3 to TGF-β1/TGF-β2 expression in wounds is considered to be a determining factor for physiological or pathological wound healing.

IL-10 is a potent anti-inflammatory cytokine that signals via two receptors: IL-10RI and IL-10RII, which has been shown to promote crosstalk between the PI3K/AKT and STAT3 pathways to inhibit fibrosis-related gene expression (74). It has been consistently demonstrated to be a major mediator in preventing fibrosis in many animal models (58). It affects ECM remodeling by downregulating collagen synthesis in skin fibroblasts (74–77), enhancing proteolytic enzyme activity to lyse the ECM, and decreasing TGF-β1 expression to prevent fibrosis (58). It also inhibits the migration of inflammatory cells such as monocytes, macrophages, and neutrophils to control inflammation (75).

The overexpression of IL-10 has been found to decrease inflammation, and create a wound site with an environment resembling those found in embryos (50). In animal models, wound sites injected with IL-10 were found to release lower levels of inflammatory mediators and reduced collagen deposition than those injected with placebo (72, 75). The addition of IL-10 to CD1 mice resulted in visually improved scar formation that appeared closer to normal skin (50). These further support the contribution of inflammation to scar formation, but the mechanisms underlying how pro- and anti-inflammatory cytokine balancing is modulated in wound healing is not fully clear.

## 4 Discussions and future perspectives

The successful application of high-resolution imaging techniques have demonstrated that the collagen structures and arrangement in scar tissue differ significantly from those of normal tissues, however, the underlying mechanisms remain unclear. The presence of pN-collagen type III has been shown to modulate skin collagen fibril diameter, but the lower collagen type III to type I ratio found in scars over that in normal skin does not conform to the thinner collagen fibrils observed in pathological scars. We speculate that the excessive and rapid synthesis of procollagen can lead to their incomplete processing, leading to procollagen molecules being only partially cleaved. The presence/accumulation of pN-collagen type I could cause steric hindrance, preventing fibrils from growing further to reach their normal dimensions. Evidence has been found that mixing collagen type I with pN-collagen type I indeed leads to the formation of thinner fibrils (15, 24). Mice with a deficiency in Meprin, a protease that cleaves propeptides from both procollagen type I and III, show thinner skin fibrils (78). The unprocessed propeptides in collagen can further enhance crosslinking, contributing to the thicker fibres in keloid scars.

N-proteinases, which are responsible for N-terminal proteptide cleavage, have been shown to exhibit maximal activity when procollagen is properly folded into a triple helix (79). Currently, it is still unclear whether rapid and excessive type I collagen formation affects triple helix formation properly and interferes with N-propeptide cleavage. It is also unclear whether inflammation affects N-proteinase expression and function. Enhancing N-proteinase expression and its activity may be targeted to improve the fibril structures in scars. However, this may only be effective before procollagens are assembled into fibrils.

Scar collagen fibers have been found to be oriented differently from those in normal skin, and the underlying molecular mechanisms remain not yet fully understood. In a healing myocardial infarcts study using engineered tissue analogs, fibroblasts have been shown to be able to remodel their surrounding collagen matrix by exerting contractile forces on the fibers in alignment with the cell’s orientation, or deposit new fibers parallel to the cell orientation (80, 81). However, the exact orientation of fibroblasts during wound healing is still unknown and requires further studies.

The current research may have overlooked the role of cross-talks between different cytokines, which could be taken as an area for further exploration. IL-1β has been shown to affect TGF-β1 instruction to human dermal fibroblasts. IL-1β alone had no effect on α-SMA formation, but it did reduce TGF-β1 stimulated myofibroblast formation, collagen type I mRNA levels and LOX activity. Moreover, it also increased collagen type III mRNA levels, and further enhanced TGF-β1 induced collagen type III mRNA levels compared to those exposed to TGF-β1 alone (82). The effects of IL-1β on TGF-β1 have been attributed to IL-1β reducing the expression of the Hedgehog transcription factor, Glioma-associated oncogene homolog 1 (GLI1). TGF-β1 stimulation upregulates GLI1 expression to facilitate fibroblast differentiation into myofibroblasts, hence its reduction inhibits myofibroblast formation (82).

It is believed that a dynamic balance of pro- and anti- inflammatory signals play a very important role in determining wound healing outcomes. Prolonged inflammation contributes to scar formation. The levels of pro- and anti- inflammatory cytokine expression have been shown to determine disease outcomes, and the injection of anti-inflammatory IL-10 lowers the levels of collagen deposition in wound healing, although one cytokine can also interfere with other cytokines’ signal transduction and hence their effects. For example, IL-6 has been shown to play an anti-inflammatory role by activating JAK/STAT signaling pathway of rheumatoid arthritis (RA) synovial fibroblasts, however, its anti-inflammatory activity was inhibited by treating cells with IL-1, which prevented STAT-dependent gene expression (83). Hence, in addition to controlling the levels of cytokine expressions, the modulation of their signal transduction can also be targeted to develop treatments against diseases. However, the current challenges here are that the molecular details of some cytokine signaling pathways are not yet fully clear, and some cytokines also share receptors (84), therefore more mechanistic studies are urgently needed.

## Author contributions

CZ: Conceptualization, Writing – original draft, Writing – review & editing. YG: Supervision, Writing – original draft, Writing – review & editing.
